# Alcohol Hangover Does Not Alter the Application of Model-Based and Model-Free Learning Strategies

**DOI:** 10.3390/jcm9051453

**Published:** 2020-05-13

**Authors:** Julia Berghäuser, Wiebke Bensmann, Nicolas Zink, Tanja Endrass, Christian Beste, Ann-Kathrin Stock

**Affiliations:** 1 Chair of Addiction Research, Institute for Clinical Psychology and Psychotherapy, Faculty of Psychology TU Dresden, Chemnitzer Str. 46, 01062 Dresden, Germany; Julia.berghaeuser@tu-dresden.de (J.B.); tanja.endrass@tu-dresden.de (T.E.); 2 Cognitive Neurophysiology, Department of Child and Adolescent Psychiatry, Faculty of Medicine, TU Dresden, Fetscherstr. 74, 01307 Dresden, Germany; Wiebke.Bensmann@ukdd.de (W.B.); Nicolas.Zink@ukdd.de (N.Z.); Christian.Beste@ukdd.de (C.B.)

**Keywords:** alcohol, cognitive effort, decision making, hangover, model-based, model-free

## Abstract

Frequent alcohol binges shift behavior from goal-directed to habitual processing modes. This shift in reward-associated learning strategies plays a key role in the development and maintenance of alcohol use disorders and seems to persist during (early stages of) sobriety in at-risk drinkers. Yet still, it has remained unclear whether this phenomenon might be associated with alcohol hangover and thus also be found in social drinkers. In an experimental crossover design, *n* = 25 healthy young male participants performed a two-step decision-making task once sober and once hungover (i.e., when reaching sobriety after consuming 2.6 g of alcohol per estimated liter of total body water). This task allows the separation of effortful model-based and computationally less demanding model-free learning strategies. The experimental induction of alcohol hangover was successful, but we found no significant hangover effects on model-based and model-free learning scores, the balance between model-free and model-based valuation (ω), or perseveration tendencies (π). Bayesian analyses provided positive evidence for the null hypothesis for all measures except π (anecdotal evidence for the null hypothesis). Taken together, alcohol hangover, which results from a single binge drinking episode, does not impair the application of effortful and computationally costly model-based learning strategies and/or increase model-free learning strategies. This supports the notion that the behavioral deficits observed in at-risk drinkers are most likely not caused by the immediate aftereffects of individual binge drinking events.

## 1. Introduction

Alcohol is a widely used, and often abused, substance that may cause a number of different adverse effects during acute intoxication, but also thereafter [1]. Especially after the consumption of larger-than-usual doses, there is a high risk of developing alcohol hangover [2], which is defined as the “the combination of negative mental and physical symptoms which can be experienced after a single episode of alcohol consumption, starting when blood alcohol concentration (BAC) approaches zero” [3]. Symptoms that are commonly reported during hangover include nausea and vomiting, headaches and stomach pains, clumsiness and weakness, tiredness and sleepiness, depressive symptoms and apathy, dizziness and confusion, as well as concentration problems [4]. Based on such recurring subjective reports and in line with studies postulating reduced workplace productivity and safety during alcohol hangover [5], it is often readily assumed that various physiological and cognitive functions are impaired during hangover. Yet, this seemingly apparent conclusion has become challenged by several studies showing that not all functional domains appear to be (equally) impaired [6]. On the physiological level, for example, it has been reported that hangover reduces performance in athletics [7] and in military contexts [8], but there are also contradictory findings suggesting that holiday activities like hiking performance do not seem to be objectively impaired in hungover individuals (even though study participants reported greater subjective exhaustion) [9]. Likewise, there are repeated reports of impaired cognition in the domains of attention and memory [6,10], which can however not be found in all studies and tasks investigating these phenomena [6,11,12]. Despite such heterogeneous findings and despite the fact that most of the tested functional domains require investing voluntary effort, which is considered to be (potentially) straining, it has never been systematically investigated whether alcohol hangover might actually reduce the ability and/or willingness to invest cognitive effort, rather than the general ability to perform a given task. Yet, this could help to explain the observed heterogeneity of effects, like why physical impairments have been reported in working contexts [7,8], but not necessarily in recreational activities [9]. Beyond this, improved knowledge about alcohol effects on engagement of effortful cognitive processes could also help to better understand phenomena like hangover-related increases in workplace absenteeism [13], or under which circumstances hungover individuals might still be able to compensate deficits by means of increased effort [14,15].

When investigating alcohol effects on the investment of cognitive effort, one can make use of the fact that behavior may be generated by relying on different strategies that vary in how much voluntary effort and control they require. This is all the more important, as both acute and chronic effects of aberrant alcohol consumption seem to strongly impair performance in tasks that require effortful cognitive top-down control, while performance is rather unaltered in tasks that require substantially less effortful automatic processes [16,17,18,19,20]. However, many of these studies typically make the participants perform both hard and easy tasks, thus confounding the findings with the factor of task difficulty (which should not be confused with effort). More importantly, the tasks used in these studies did typically not provide the participants with the possibility to choose a strategy for themselves, or to arbitrate between more and less straining strategies. Investigating the arbitration between effortful top-down controlled “model-based” behavior and less demanding “model-free” behavior is not only of scientific interest, but also of clinical relevance: A better understanding of the mechanisms underlying intra- and inter-individual differences can elucidate the behavioral and psychological changes that have been associated with problematic drinking patterns like binge drinking [21,22] and shown to drive and maintain alcohol use disorders (AUDs) [16,23,24,25].

In the framework of reinforcement learning, model-based and model-free learning can be distinguished from each other as two classes of methods. Model-based learning uses an internal model of the environment and enables us to take appropriate actions through planning, which is based on that model and on the expected outcomes of the available choice options. While this is computationally demanding, model-based learning can quickly incorporate and adjust to changes in environmental structures or in outcomes and is thus associated with adaptive and flexible (goal-directed) behavior [26]. In contrast to this, a model-free strategy does not use a model of the world. Instead, model-free behavior uses prediction errors to learn the (outcome) values of the available choice options. Those values are stored in scalar quantities and can be easily accessed so that model-free learning is computationally cheap. The downside of this strategy is that changes in the environment or in outcomes can only slowly be incorporated in the values of the choice options through trial-end-error learning, which makes model-free learning less adaptive [26,27].

The arbitration between more or less demanding cognitive-behavioral strategies can be assessed with the Markov decision task (also called two-step task), which was specifically designed to disentangle model-based and model-free learning strategies [28]. This task requires participants to make two successive decisions, which lead to an outcome (differently sized gains or losses) in the end of each trial. The outcomes change throughout the course of the task, which necessitates constant updating (learning). Crucially, the first-level decision leads to one of two second-level states, and therefore to different associated choice options, with certain probabilities. This transition structure of the task can be used for the model-based learning strategy or can be neglected in case of model-free learning, which allows to computationally distinguish the two strategies [27,28].

We applied the two-step task and subsequent parameter modeling to *n* = 25 healthy young men, who participated in a within-subject experimental design [11] where they were tested once sober and once hungover (i.e., after a night of experimentally induced drinking). Our hypotheses were based on the findings that AUD patients and heavy binge drinkers (BD) have previously been shown to demonstrate significant reductions in effortful controlled model-based cognitive strategies, thus inducing an imbalance between model-free and model-based behavior (as compared to healthy controls) [21,25]. While there is broad consensus that AUD patients shift from model-based to model-free behavior [23], it should however be noted that this pattern could not be completely observed in all studies investigating the phenomenon. For example, Voon et al. did not find differences between abstinent AUD patients and healthy matched controls [29], while Sebold et al. could not reproduce their initial findings of selectively impaired goal-directed functions [25] in a larger AUD sample [30]. Furthermore supporting our hypotheses that alcohol might shift the balance between model-based and model-free behavior, it has been demonstrated that the BD-associated imbalance in favor of rather effortless model-free behavior seems to normalize as the time since the last binging event increases [21]. Lastly, increased perseveration tendencies (i.e., reduced cognitive flexibility) have been observed in BD, but not in case of abstinent AUD patients [21,29,31]. Thus, increased perseveration tendencies might also potentially be found during alcohol hangover. Therefore, we hypothesized that alcohol hangover could induce qualitatively similar effects, albeit probably to a lesser degree. The investigation of AUD patients and BDs alone does not allow for any conclusions about whether the shift from model-based to model-free behavior observed in these groups reflects premorbid deficits. Yet, the lack of such “premorbid” changes in control participants with a positive family history of AUD [32] as well as in otherwise healthy, young BDs [33] suggests that this might not be the case and that this behavioral shift is rather a consequence of excessive alcohol consumption. Against the background that habitual binge drinking might induce a shift from model-based to model-free behavior that can still be observed after the end of an acute binge-like intoxication [21], we hence hypothesized that this was also the case during the hangover following a single binge drinking episode.

## 2. Materials and Methods

### 2.1. Participants

Healthy young men aged 18–30 were recruited via flyers and online ads at the local university (TU Dresden). In order to be included in the sample, all participants underwent an extended telephone screening, during which their somatic, neurological, and psychiatric well-being, as well as their alcohol consumption were assessed with the help of a semi-structured interview by experienced neuropsychologists. They had to report to have normal or corrected-to-normal vision, be free of psychiatric and neurologic disorders, as well as somatic diseases (especially those affecting the gastrointestinal tract, liver, and kidneys). Likewise, they had to report not taking any medication or illicit drugs either regularly, or during their participation in the study (including a sufficient number of preceding days in case the metabolism of a given substance took more than 18 h). With respect to alcohol consumption habits, we required all included participants to have scores between 2 and 19 points in the Alcohol Use Disorder Identification Test (AUDIT) [34]. Additionally, they were required to have voluntarily engaged in binge drinking (defined as consuming 8 or more standard units of alcohol on a single evening) between 13 and 150 times in the past year and to recall at least one event within the past year when they were markedly drunk (defined as experiencing alcohol-induced gait, motor, or speech impediments). Individuals who had less than 2 points in the AUDIT or drank less than these lower limits were excluded in order to minimize the risk of including participants who might become unwell after drinking the alcohol dose we experimentally administered to induce intoxication and subsequent hangover. We further excluded individuals who had more than 19 points in the AUDIT (as scores of 20 points or more “clearly warrant further diagnostic evaluation for alcohol dependence” [34]), drank more than our pre-defined upper limits (as binge drinking on 3 or more days a week shows that binge-like alcohol consumption is no longer limited to social drinking on weekends), and/or reported having at least weekly alcohol-induced memory problems and/or at least near-daily failures to fulfill routine tasks that were expected of them (as this would have indicated a high and likely clinically relevant degree of alcohol-related cognitive dysfunction). In sum, these upper thresholds were implemented to minimize the likelihood of including individuals with strong alcohol tolerance and a high risk for AUD. The study was approved for males only by the ethics committee of the Faculty of Medicine of the TU Dresden, Germany (EK293082014). All participants provided written informed consent at the start of each study appointment while (still) sober. They received a compensation of 80€ for study participation. 

There were no previous studies investigating the size of hangover effects in Markov decision tasks, but studies on other cognitive control domains reported effect sizes between f = 0.32 and f = 0.6 for their reported hangover effects in comparable within-subject study designs [11,17,35]. Based on this, we estimated the required sample size for two repeated measures sessions (sober vs. hungover) and five relevant measures (MF-score, MB-score, final score, ω, π) at an alpha error probability of 5% and a power of 95% for an estimated medium effect size of f = 0.30 (assuming a default inter-correlation of 0.5). This yielded a required sample size of *n* = 23. Based on this initial sample size estimation, *n* = 25 subjects matching all of the criteria detailed above were eventually included in the sample and underwent experimental testing as well as statistical analyses. Please note that the sample used in this publication strongly overlaps with that of a previous publication, which investigated alcohol hangover effects on attentional processes during varying conflict loads in a prime and flanker context [11]. 

### 2.2. Experimental Design

Importantly, we used the same study design as already reported in our previous publication [11]. In short, each participant was invited to the lab for three different appointments the order of which was balanced across the sample so that half of the participants first performed the paradigm sober and then hungover, while the other half first performed the paradigm hungover and then sober. Participants could not start with any of their appointments unless they were entirely sober at the start of each appointment. The required breath alcohol concentration (BrAC) of 0.00‰ was controlled using the breathalyzer “Alcotest 3000” following the instructions by the manufacturer (Drägerwerk, Lübeck, Germany). Participants were further required to refrain from using legal stimulants like coffee, taurine, or guarana in the three hours preceding each appointment and to eat a full dinner before participating in the intoxication appointment. 

On both the sober and the hangover appointment, the participants rated their subjective hangover symptoms on a Likert scale (see Section 2.3 for details) and then performed a total of four conceptually unrelated behavioral tasks. The results of two of these tasks have been previously been published [11], and the results of the third task, which assessed mental rotation and response inhibition, have not been published or submitted anywhere, as of yet. The task reported in this study was always conducted last (i.e., approximately 60–75 min after the start of the appointment). The sober appointments were conducted on weekdays and between 2 and 7 days apart from the hungover appointments, which were always conducted on Saturday or Sunday (starting time between 09:00 and 11:00) after a previous night of experimentally induced alcohol intoxication. These intoxication appointments took place on Friday or Saturday, starting at 20:00. For each intoxication appointment, we invited between 2 and 6 subjects to the lab. They were asked to fill in a sociodemographic questionnaire and then consume an individually determined amount of 2.6375 g of alcohol per estimated liter of total body water (TBW), which was determined with an equation by Widmark [36] and Watson et al. [37]. The details of equation as well as the protocols used to document drinking can be found in our previous publication [11] and in the data sheet provided in the Supplementary Materials. In line with recommendations from previous experimental studies [2,38,39,40,41], the administered amount of alcohol was expected to result in a mean peak intoxication of ~1.2 ‰ on the full stomach we asked participants to have (i.e., at a resorption deficit of ~40%), and no more than 1.6‰ on an empty stomach, which we asked participants to avoid (i.e., at a resorption deficit of ~20%). Due to the ratio of TBW and administered alcohol, it was physically impossible to exceed a peak intoxication of 2.0‰ (i.e., at a hypothetical resorption deficit of 0%). Additionally, the experimenters did not issue more than half of a participant’s drinks within the first hour of drinking so that participants were kept from consuming the entire amount at once (consumption typically took 2–3 h). Participants got their drinks from the experimenters and could choose whether they wanted 200 mL red wine (9.5 Vol % equaling 15 g of alcohol) or 50 mL brandy (36 Vol % equaling 14 g of alcohol) with each refill. These two drinks were chosen for their comparatively high congener content, which is thought to increase hangover severity (as compared to beverages with lower congener content, like vodka or white wine) [42,43,44]. Drinks could be mixed with caffeine-free softdrinks (coke, orange lemonade, ginger ale) and ice cubes. Participants were further provided with unlimited access to snacks (chips and wine gum) and tap water, the consumption of which were not monitored. They were furthermore allowed to smoke. Participants were free to socially interact, listen to music, play board and card games, or table soccer during the intoxication appointment. 30, 60, 90, and 120 min after the individual end of their consumption, participants were asked to provide BrAC measurements. They were then sent home via taxi around 1:30 to 02:00 in the morning (given decreasing BrAC values and no clouded awareness and/or major motor impairments). They were invited to come back the following day at either 09:00 or 10:30 for their hangover appointment. This was done for two reasons: Firstly, we wanted to test the participants as soon as possible after reaching the sobriety criterion of 0.00‰ because hangover-associated cognitive deficits of social drinkers might be most pronounced at this time point [45] (if they failed to reach this criterion at the originally scheduled time, they were asked to wait until BrAC had returned to 0.00‰). Secondly, it has been recommended to standardize sleep time in experimental hangover induction [41,46] as reduced sleeping time could be associated with more severe hangover symptoms (although reduced sleep time and quality are of course also directly associated with alcohol intoxication itself) [42,44,47,48,49]. Yet still, alcohol effects on sleep do not seem to necessarily mediate hangover effects on cognitive performance [40,44]. Lastly, it should be noted that while we experimentally standardized sleeping times across the sample for optimal comparability across participants, both the time at which participants could go to bed and the estimated average sleeping time were oriented towards normal behavior in young healthy social drinkers, as previously reported in a study with a naturalistic study design (in that study, average drinking started between 20:06 and 21:06, average bedtimes were between 02:49 and 03:18 am, and the average sleep duration was between 05:36 and 05:58 h) [47].

### 2.3. Questionnaires

At the start of the intoxication session and before alcohol administration, subjects provided sociodemographic information. At the beginning of both the sober and hangover session, participants were asked to rate the subjective severity of 22 hangover symptoms suggested by van Schrojenstein Lantman et al. [4,50] on an 11-point Likert-scale ranging from 0 (no symptoms) to 10 (extreme symptoms). Importantly, participants were asked to truthfully rate the severity of each symptom irrespective of whether they had consumed alcohol the night before or attributed their symptoms to alcohol consumption. Furthermore, subjects reported the hours of sleep during the previous night.

### 2.4. Two-Step Decision-Making Task

**Figure 1:**
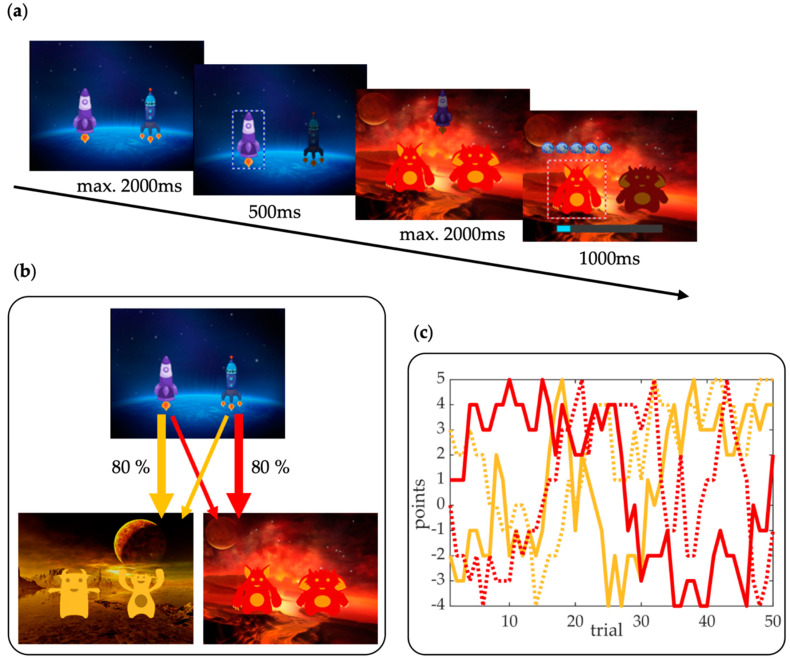
Two-step decision-making task. (**a**) An exemplary trial sequence as well as the trial timing are depicted: At the first stage, two spaceships were presented. Participants indicated their response choice via a button press, followed by a transition two the second stage. Two aliens represented second-stage choice options and participants made their second response choice via another button press. Response choices were indicated by boxes around the respective spaceship/alien and trial outcomes are indicated by blue spheres (space treasure) representing the number of gained points (+5 shown) or pink spheres (antimatter) representing the number of lost points (not shown). The response time limit was 2 s for each of the two choices. According to the transition structure (**b**), a transition could either be common (80% probability) or rare (20% probability). After the second stage response, the outcome was presented. (**c**) The outcomes (+5 to −4 points) of the four choice options are presented for the first 50 trials. Each line represents a second-stage choice option (alien) for the yellow and red planet, respectively.

In order to investigate whether alcohol hangover reduced cognitively effortful model-based behavior and/or increased the less costly model-free behavior, we used a modified two-step decision-making task based on Daw et al. [28] and Kool et al. [27], which was embedded in a space game. Each trial consisted of two sequential decisions that led to a final outcome. As can be seen in Figure 1, two different spaceships were presented in the beginning of each trial to represent the choice options at first stage. The spaceships were associated with a transition probability of 80% (common transition) to reach one of two planets, and a transition probability of 20% (rare transition) to reach the other planet. These planets indicated the second stage options. At the second stage, two new choice options were presented in the form of different aliens. The subjects were told that the aliens mine in “space mines” where they could find either treasures (representing positive outcomes), or antimatter (negative outcomes), or nothing. The outcomes for each of the four second-level choice options slowly changed throughout the task. Therefore, the value of each option had to be constantly updated. The outcomes ranged from −4 to +5 points and magnitudes were slowly drifting according to a Gaussian random walk: The outcomes for each of the four options at second stage were calculated independently so that they ranged from 0 to 1 and slowly changed with a drift rate of 0.2. The resulting scores were then transformed into points. The transition distribution and reward distribution were the same for all subjects. Those distributions were simulated beforehand in order to ensure that model-based engagement would lead to higher final scores (for details, please see section “Simulation of Transition and Reward Distribution” in the Supplementary Materials). In other words, this made sure that the more costly model-based strategy always paid off more than the model-free strategy. At the end of each trial, a bar was presented to indicate the current total score. We modified the original two-step task by Daw et al. [28] in several ways based on simulation results of Kool et al. [27] in order to allow for a stronger relationship between model-based learning strategy and reward payoff: Firstly, we used a simpler, more distinguishable transition probability of 80:20 (instead of the original 70:30) to reduce rare transition trials, which was intended to reduce the trade-off between pay-off and cognitive costs. Secondly, we increased the drift rates of second stage outcomes and used a broader range of reward probabilities (Gaussian random walk: M = 0, SD = 0.20, reflecting boundaries = [0 1] vs. originally: Gaussian random walk: M = 0, SD = 0.025, reflecting boundaries = [0.25 0.75]) to induce faster changes of rewards, which was intended to reduce the possibility of easy adaptations of model-free learning and thereby increase the relative advantage of model-based learning. Thirdly, we used points instead of binary probabilistic outcomes to increase the information gain of each trial and thus reduce the necessity to integrate information over several outcomes per choice option. Taken together, these modifications should have resulted in a higher pay-off for the more cognitive costly model-based strategy. The task consisted of 250 trials, which were divided into two equally sized blocks. The main goal of the subjects was to collect as much treasure (points) as possible. The screen position (left or right) of the two choice options was randomized across trials for stimuli at both stages. If no response was made via button press on a standard keyboard within the 2 s response limit, participants received a penalty loss of 5 points and the trial was repeated. Prior to the main task, subjects received detailed instructions and tutorials, including 25 practice trials to familiarize them with the task. The task was presented with Presentation software (Neurobehavioral Systems Inc., Berkeley, CA, USA). Trial timing is illustrated in Figure 1a. We used different planet and alien stimulus sets at the two appointments in order to minimize carry-over effects between the sober and the hungover appointment. 

Importantly, the task allows the detection and dissociation of model-free vs. model-based decision-making behavior. This becomes especially apparent after rare transitions that ultimately lead to high rewards: In those cases, an entirely model-free agent would repeat the choices that resulted in this reward (i.e., the agent would choose the same action/spaceship again) in accordance with basic reinforcement principles, which state that the probability to choose an option again is higher when this option was previously rewarded. In contrast, a model-based agent would take into account the model of the task, i.e., the knowledge of the transition probabilities between stages. In that case, the probability to choose the same action again would be much lower, because the valuation system would take into account that the other option has a much higher probability to lead to the promising second stage option. Therefore, a model-based agent would likely switch the first stage choice under circumstances of high rewards after rare transitions. 

Following this logic, first stage choice behavior can be utilized to determine and distinguish signatures of model-based and model-free learning. For that purpose, stay probabilities can be computed, i.e., the probability to choose the same first stage option again, as a function of previous outcome (win or loss) and transition type (common or rare). Those probabilities can be used to calculate a model-free score (MF-score) and a model-based score (MB-score) for each subject in order to analyze the reliance on the respective learning systems [25]. The MF-score indicates the pure influence of previous reward on the first stage choice pattern:

MF-score = (Stay_win common_ + Stay_win rare_) − (Stay_loss common_ + Stay_loss rare_).

In contrast, the MB-score reflects the interaction effect of previous reward and transition type on stay probability, and thus the consideration of the model of the task for first stage choices:

MB-score = (Stay_win common_ + Stay_loss rare_) − (Stay_win rare_ + Stay_loss common_).

Both scores consider choice behavior with regard to the previous trial, but ignore performance throughout the whole task, which can be provided by computational modeling accounts.

The dual-system reinforcement-learning model is an established computational model for the task we used. It assumes a mixture of model-based and model-free learning strategies [27,28]: During the course of the task, expected values (Q-values) will be learned for each choice option (*a*) in each state (*s*) at the two stages (*i*). The model-free value (*Q_MF_*) is updated at each trial (*t*) according to a state-action-reward-state-action, or SARSA(*λ*) temporal difference learning algorithm [27,51]. After each action, an update takes place to calculate a new estimate of the value of the chosen option based on the agent’s experience. The general updating rule is:

(1) $$Q_{MF}\left(s,a\right)=Q_{MF}\left(s,a\right)+\alpha \delta_{i,t}$$

where *α* denotes the learning rate and *δ* is the reward prediction error:

(2) $$\delta_{i,t}=r_{i,t}+Q_{MF}\left(s_{i+1,t},a_{i+1,t}\right)-Q_{MF}\left(s_{i,t},a_{i,t}\right)$$

with *r* denoting the received reward. The learning rate determines to which extent the new information provided by the reward prediction error is incorporated in the value estimate. At first stage, the reward prediction error is solely driven by the Q-value of the option that is chosen at second stage, since no reward is delivered at the first stage:

(3) $$\delta_{1,t}=Q_{MF}\left(s_{2,t},a_{2,t}\right)-Q_{MF}\left(s_{1,t},a_{1,t}\right).$$

At the second stage, the reward prediction error is driven by the received reward, since no third stage is available:

(4) $$\delta_{2,t}=r_{2,t}-Q_{MF}\left(s_{2,t},a_{2,t}\right).$$

The Q-values for both stages are updated at the end of each trial. For the update of the first stage model-free Q-value, a decay-rate parameter for eligibility traces (*λ*) is used to additionally down-weight the second stage prediction error:

(5) $$Q_{MF}\left(s_{1,t},a_{1,t}\right)=Q_{MF}\left(s_{1,t},a_{1,t}\right)+\alpha \lambda \delta_{2,t}.$$

In contrast to this, the model-based strategy for the first stage update considers the transition probability *P* (model of the environment) between stages and combines this knowledge with the values of second stage options. It is assumed that the transition probability is fixed and known to the agent:

(6) $$Q_{MB}\left(s_{1,t},a_{j}\right)=P(s_{2A}|s_{1},a_{j})\;max\;Q_{MF}\left(s_{2A,t},a\right)+P\left(s_{2B}|s_{1},a_{j}\right)max\;Q_{MF}\left(s_{2B,t},a\right)$$

where *j* denotes the index of the first stage choice options and $s_{2A}$ and $s_{2B}$ the two different states at the second stage. At the second stage, the updating rule for values is the same as for the model-free strategy.

To select an action at the first stage, the model-free and model-based Q-values are combined and weighted by the parameter *ω*:

(7) $$Q_{net}\left(s_{1},a_{j}\right)=\omega Q_{MB}\left(s_{1},a_{j}\right)+\left(1-\omega \right)Q_{MF}\left(s_{1},a_{j}\right).$$

A low weighting parameter (*ω* < 0.5) indicates a stronger reliance on the model-free strategy, whereas high values (*ω* > 0.5) indicate a stronger influence of the model-based strategy. At second stage, both learning strategies use the model-free Q-value for action selection. 

The probability to choose an action at each stage is computed according to a sofmax rule:

(8) $$P\left(a_{i,t}=a|s_{i,t}\right)=\frac{exp\left(\beta \left[Q_{net}(s_{i,t},a)+\pi \cdot rep\left(a\right)\right]\right)\;}{\sum_{a\prime }exp\left(\beta \left[Q_{net}(s_{i,t},a\prime )+\pi \cdot rep\left(a\prime \right)\right]\right)}$$

where the inverse temperature *β* determines the stochasticity of the choices. Higher *β* values indicate that the agent is more likely to choose the action with the highest Q-value (i.e., high expected outcome) and lower *β* values indicate a tendency towards random choice (i.e., that the agent’s decisions are less determined by this learning strategy). Additionally, a choice “stickiness” parameter *π* was included, which was multiplied with an indicator variable rep(a) that indicates whether the same action was chosen again, or not. This parameter indicates perseveration (*π* > 0) or switching (*π* < 0) tendency. Lastly, optimal choice rates were separately calculated for each stage [52]. These rates reflect whether decisions were made in favor of the option with the higher Q-value or not, and thus reflect the probability of choosing the optimal option. The model fitting was conducted with Matlab 2018b (The MathWorks, Inc., Natick, MA, USA), with empirical priors using Sam Gershman’s mfit toolbox to find the maximum a posteriori parameter estimates [27,53].

In summary, the MF- and MB-scores represent different influences of simple reinforcement learning vs. effortful goal-directed computation for action selection which is based on the experience of the previous trial. Whereas the computational model considers choice behavior over the course of the whole task, the weighting parameter omega (*ω*) indicates the relative contribution of model-free and model-based strategies to decision-making and thus the extent of cognitive investment. The choice stickiness parameter *π* indicates the arbitration between behavioral perseveration and switching. Lastly, *β* values indicate to what degree the participant is likely to choose the response that is associated with the highest expected outcome. Together with the optimal choice rate, these variables reflect whether decisions were made in favor of the option with the higher Q-value. Finally, the sum of all collected outcomes (final score) and reaction times for choices at first and second stage may be used to compare the overall performance.

### 2.5. Statistical Analyses

To compare task performance between the sober and hangover session, we used the Bayesian procedure for related samples provided by SPSS Statistics 25 (IBM Corp., Armonk, NY, USA), which computes a traditional (paired samples) *t*-test and the Bayes Factor (BF). For this, we used default settings (Adaptive Gauss-Lobatto Quadrature approach, Tolerance = 0.000001, maximum iterations = 2000) with a noninformative prior (diffuse prior distribution). To check the normality assumption, we used the Shapiro-Wilk-Tests and conducted additional non-parametric tests, whenever necessary. 

The BF indicates the ratio of the data likelihood given the null hypothesis versus the data likelihood given the alternative hypothesis: A value above one indicates (more) relative evidence for the null hypothesis whereas values below one indicate (more) relative evidence for the alternative hypothesis. Values above three are considered as positive evidence for the null hypothesis, i.e., no difference between the sober and hangover session [54].

Since we were mainly interested in hangover-associated differences in model-based and model-free learning, we analyzed MB-scores and MF-scores, which were calculated for each subject and session, as well as the weighting parameter ω. To analyze perseveration tendencies, we focused on the choice stickiness parameter π. All other parameters provided by the computation model were analyzed in an exploratory fashion. The Bayesian information criterion (BIC) was used to verify comparable model fit between sessions. To further examine whether the participants had based their decisions on hybrid Q-value estimation to a similar degree in both of their sessions, we analyzed their optimal choice rates for each stage [52]. Finally, we used the sum of all collected outcomes (final score) and reaction times for choosing at first and second stage to compare the overall performance.

Given that we balanced the order of the two appointments across participants, used two different task versions/stimuli on the first and second appointment, and further randomized stimulus positions on the screen for each trial, we did not anticipate any confounding effects of appointment order. For this reason, appointment order was not included as a factor in any of the analyses presented in the results section but add-on analyses of this factor can be found in the section “Investigation of Hypothetical Task Order Effects” of the Supplementary Materials. 

The raw behavioral data as well as the analyzed data (including the syntax) can be accessed at https://osf.io/vzpn3/.

## 3. Results

### 3.1. Sample Characteristics and Intoxication Procedure

The included participants were on average 21.5 years old (SD = 2.3; range 18–27), 183.4 cm tall (SD = 7.0; range 167–198), and weighed 80.5 kg (SD = 11.6; range 56.5–96.5). This resulted in an average individual alcohol amount of 432.0 mL brandy (SD = 39.01; range 349–497) at 36 Vol. %. Participants took on average 174.4 min (SD = 28.3; range 115–230) to consume the alcohol. The mean BrAC was 1.17 ‰ (SD = 0.23; range 0.75–1.63) 30 min after the end of consumption, 1.09‰ (SD = 0.22; range 0.65–1.51) 60 min after the end of consumption, 1.04 ‰ (SD = 0.17; range 0.65–1.41) 90 min after the end of consumption, and 0.94 ‰ (SD = 0.14; range 0.67–1.16) 120 min after the end of consumption.

As would have been expected from the study design, participants reported a shorter average sleep duration in hangover session (mean = 6.05 h; SD = 0.83; range 4.50–8.00) than in the sober session (mean = 8.10 h; SD = 1.39; range 5.50–10.00). Hence, our participants slept approximately two hours less before the hungover appointment than before the sober appointment. Of note, this is very similar to the hangover-associated 1 h and 50 min sleep reduction reported in a previous, naturalistic study by Hogewoning et al. (where hungover participants had slept 7 h and 26 min on sober nights and 5 h and 36 min on hungover nights) [47]. Given that none of the task-relevant behavioral and estimated measures worsened during hangover (for details, please refer to the following text sections), there was however no need to control for the shorter sleeping time before the hungover session.

Based on the recruitment criterion that all participants had to have some degree of binge drinking experience in order to minimize the risk of severe adverse effects during alcohol administration, the mean AUDIT score of the sample was 10.1 points (SD = 2.8; range 4–16). Out of the *n* = 25 participants, *n* = 19 had scores between 8 and 15 points, which has been linked to hazardous alcohol use that does however not require clinical intervention [34]. Only *n* = 1 participant had a score of 16, which is the lower boundary for “brief counseling and continued monitoring” recommended by WHO guidelines [34]. Yet, none of the participants obtained a score of 20 or higher and none of the participants met the criteria for the diagnosis of an AUD according to the International Classification of Diseases (ICD-10). The subjective ratings for overall hangover severity and the severity of individual hangover symptoms are presented in Table 1. 

### 3.2. Two-Step Decision-Making Task

**Figure 2:**
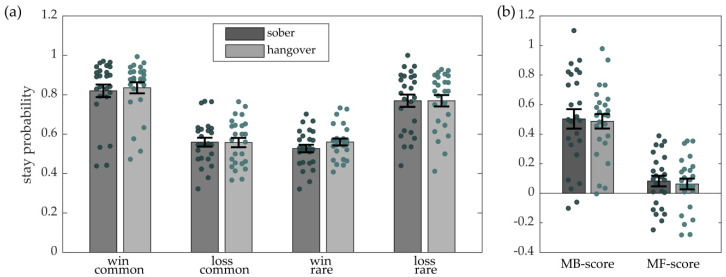
First stage choice behavior. Dots indicate values of individual participants and bars indicate group means with error bars depicting the standard error of the mean. (**a**) Stay probability (choosing the same options as in the previous trial) for win and loss trials as a function of transition (common vs. rare). The sober session is depicted in dark grey and the hangover session is depicted in light grey. (**b**) Model-based score (MB-score), reflecting the interaction between outcome and transition type, and model-free score (MF-score), reflecting the main effect of outcome, for the sober session (dark grey) and the hangover session (light grey).

Descriptive statistics are shown in Table 2 and Figure 2 shows the first stage choice behavior for the sober and hangover session.

There was no significant difference between the sober and hangover session with respect to either MB-score (*t*_(24)_ = 0.38, *p* = 0.80) or MF-score (*t*_(24)_ = 0.38, *p* = 0.71). Bayesian analyses indicated positive evidence in favor of the null hypothesis, i.e., the assumption that the MB-score (BF = 6.30) and the MF-score (BF = 6.05) did not differ between the sober and hangover session. This suggests that the degree of model-based and model-free learning was not changed by alcohol hangover.

With respect to the overall task performance, we observed that participants earned comparable cumulative points at the end of the task (final score). These outcomes did not significantly differ between sessions (*t*_(24)_ = −0.11, *p* = 0.91) and Bayesian analysis provided positive evidence in favor of the null hypothesis (BF = 6.46). The reaction times at the first stage and at the second stage did also not significantly differ between the sober and hangover session (first stage: *t*_(24)_ = 0.11, *p* = 0.91; *Z* = −0.23, *p* = 0.82; second stage: *t*_(24)_ = −0.53, *p* = 0.60). Again, Bayesian analyses provided positive evidence in favor of the null hypothesis, i.e., no difference between the sober and hangover session in response latency (first stage: BF = 6.46; second stage: BF = 5.69). These findings suggest that neither overall task performance, nor response speed are modulated by alcohol hangover.

Table 3 shows all estimated parameters based on the hybrid dual-system reinforcement-learning model. The model fit by means of BIC did not statistically differ between sessions (*t*_(24)_ = −0.28, *p* = 0.79, BF = 6.27). In the sober session, the average BIC was 504.07 (SEM = 21.48) and in the hangover session, the average BIC was 508.72 (SEM = 18.12). At the first stage, subjects reached optimal choice rates with an average choice rate of 0.66 (SEM = 0.03) in the sober session and with an average choice rate of 0.70 (SEM = 0.03) in the hangover session. Choice rates did not significantly differ between sessions (*t*_(24)_ = −0.83, *p* = 0.42; *Z* = −0.72, *p* = 0.48). At the second stage, the average optimal choice rate was 0.77 (SEM = 0.02) in the sober session and 0.76 (SEM = 0.03) in the hangover session. Like for the first stage, the second stage choice rates did not significantly differ between sessions (*t*_(24)_ = 0.33, *p* = 0.75; *Z* = −0.69, *p* = 0.50). Bayesian analyses provided positive evidence in favor of the null hypothesis (no difference between the sober and hangover session) for optimal choice rates at the first stage (BF = 4.68) and at the second stage (BF = 6.17). Therefore, both BIC and optimal choice rates indicate that participants similarly applied the hybrid Q-learning model in both sessions. In this context, please note that the decision process is assumed to include some randomness. With regard to the dynamic task environment (slowly changing rewards), it is reasonable that subject explored the other choice option from time to time, which is also reflected in the optimal choice rates. In such a dynamic environment, even perfectly adjusted behavior could not yield choice rates of (or close to) 1.

Most importantly, we found no significant difference between the sober and hangover session in the weighting parameter *ω* (*t*_(24)_ = −0.48, *p* = 0.63; *Z* = −1.39, *p* = 0.17). Further supporting this, Bayesian analyses yielded positive evidence for the null hypothesis (no difference between sessions; BF = 5.81), indicating that the balance between model-based and model-free learning was not affected by hangover status. This null finding is in accordance with the results of the MB-score (no session effect) and provides evidence for unaffected goal-directed learning in the context of this task. 

The choice “stickiness” parameter *π*, which indicates a perseveration tendency in case of values above zero, did also not differ between the sober and hangover session (*t*_(24)_ = −1.43, *p* = 0.17), but the obtained BF of 2.52 provided only weak evidence in favor of the null hypothesis.

An exploratory analysis of the learning rate α revealed no significant differences between the sober and hangover session (*t*_(24)_ = 0.73, *p* = 0.47; *Z* = −1.39, *p* = 0.17). We also found no significant difference between sessions with respect to the inverse temperature *β* (*t*_(24)_ = 0.64, *p* = 0.53), which represents the randomness of decisions, i.e., the reliance on Q-values in decision-making. Likewise, the decay-rate parameter λ did not statistically differ between the sober and hangover session (*t*_(24)_ = −0.48, *p* = 0.64; *Z* = −0.55, *p* = 0.58). Bayesian analyses provided positive evidence for the null hypothesis (i.e., no difference between the sober and hangover session), for learning rate *α* (BF = 5.03), inverse temperature *β* (BF = 5.33), and decay-rate parameter *λ* (BF = 5.82). Thus, our exploratory analyses suggest that none of these parameters seems to be modulated by alcohol hangover.

### 3.3. Add-On Analyses of Alcohol Consumption Habits

Given that a recent study found acute alcohol intoxication effects on model-based behavior to be modulated by drinking problems (as assessed with the AUDIT) [55], we ran exploratory add-on analyses to investigate whether AUDIT scores correlated with any of the functionally relevant descriptive or estimated parameters in the sober and/or hungover session. As can be seen in Table 4, we did not find any significant correlation in either the sober or the hungover session. Bayesian analyses (default settings for Bayesian Pearson Correlation: Tolerance = 0.0001, maximum iterations = 2000; uniform prior; Jeffreys–Zellner–Siow Bayes Factor) provided positive evidence in favor of the null hypothesis (no relationship between AUDIT and task performance) for MF-score, MB-score, weighting parameter ω, and choice stickiness parameter π; and weak evidence in favor of the null hypothesis for the final score (earned cumulative points at the end of the task). We therefore refrained from using the AUDIT as a control variable/covariate in any of the main analyses. 

## 4. Discussion

Aberrant alcohol consumption has repeatedly been demonstrated to be associated with negative cognitive, affective, and behavioral consequences [1]. While the effects of acute intoxication and long-term abuse are comparatively well-researched, much less is known about the cognitive and behavioral effects of alcohol hangover. In this study, we used an experimental cross-over design to test the hypothesis that alcohol hangover decreases model-based and increases model-free behavior. A total of *n* = 25 healthy young men were tested with a two-step task. Each participant was tested once sober and once hungover, i.e., after having consumed a standardized amount of alcohol in an experimental setting. Several behavioral and computational modeling parameters were then compared across the two sessions. Our study motivation and hypotheses had been based on several studies showing that alcohol seems to have much stronger detrimental effects on goal-directed/model-based processes that require high levels of cognitive effort, than on model-free processes which typically require substantially lower levels of effort. This observation has repeatedly been made in the context of acute, binge-like intoxication levels [18,19,56,57,58], and in the context of AUD [16,20,23]. In social BDs, who do not fulfil enough criteria for an AUD diagnosis, results are generally more mixed, but there are also repeated reports of impairments in the domain of goal-directed (executive) functions [21,22]. With respect to the arbitration between goal-directed and habitual behavior, both AUD and BD have been linked to reductions in effortful controlled model-based cognitive strategies [21,25]. At least in BD, this imbalance seems to normalize as the time that has passed since the last binging episode increases [21]. Moreover, perseveration tendencies seem to be altered in BD [21,31]. Based on these findings, we had hypothesized that alcohol hangover might induce qualitatively similar effects, albeit probably to a lesser degree. 

The employed two-step decision making task based on Daw et al. [28] and Kool et al. [27] allows for the quantification of model-based and model free behavior by contrasting first stage stay probabilities in case of all combinations of gain/loss and common/rare transitions on the one hand and by estimating individual parameters of task performance with a computational model on the other hand. The underlying logic is that model-free behavior is solely based on previous rewards/losses and does not consider transition probability, which makes it computationally cheap, but also rather inflexible. Following this strategy, first stage choices are repeated whenever that choice has been rewarded, and switched when that choice has been not rewarded or has been punished. In contrast to this, model-based choices should additionally account for transition probabilities, which makes it computationally more demanding and effortful, but also more flexible and adaptive. Following this strategy, first stage choices tend to be repeated whenever a choice has been rewarded on a common transition or punished on a rare transition, and switched when a choice has been rewarded on a rare transition or punished on a common transition.

Even though the experimental induction of hangover was effective (as demonstrated by significant increases in 21 out of 22 assessed hangover symptoms, as well as overall hangover severity [59]), we did not find evidence for any hangover effects in the MF- and MB-score, as well as in the computationally deduced weighting parameter ω (which represents the balance between the two strategies), or in the overall outcome (obtained score). Instead, Bayesian analyses provided positive evidence that there was likely no difference between the sober and the hungover session. The lack of response time effects further suggests that the application of goal-directed strategies was not maintained at the cost of a speed-accuracy tradeoff. The BIC parameter, which allows to compare the model fit across sessions, further suggested that the observed comparability across sessions was not caused by differences in the goodness of the model fit. Likewise, we found no evidence for increased perseveration tendencies (*π*) during hangover. Subsequent Bayesian analysis failed to provide conclusive evidence for either hypothesis, but still favored the null hypothesis over the alternative hypothesis at an anecdotal level. Hence, all of our findings are in favor of the assumption that alcohol hangover does not alter the balance between model-based and model-free learning strategies, or increase perseveration tendencies. Still, it would be commendable to also investigate other potential facets of alcohol hangover effects on automatic and/or habitual behavior with other promising new paradigms [60,61]. Add-on exploratory analyses further showed that there were also no hangover effects on the learning rate (*α*), the randomness of decision-making (*β*), or the down-weighing of previous experience (*λ*). 

It should however be noted that the weighting parameter omega was numerically higher than in other studies with healthy young samples [32,62,63], which indicates a stronger preference for the model-based learning strategy in the investigated sample/applied task. This could be due to the manipulations in task administration (e.g., more prominent transition probabilities as well as usage of simulated distributions for outcomes and transitions), which facilitates the application of model-based, computationally demanding strategies [27]. A higher reliance on a model-based system is typically found when high incentives shift the cost-benefit-arbitration in favor of a computationally costly strategy [64,65]. Given this strong preference for model-based over model-free strategies in both sessions, we can assume a general willingness to exert cognitive effort and thereby exclude the possibility that the lack of hangover effects could be due to a lack of overall motivation to perform the task as instructed. It however remains an open question whether high intrinsic motivation or changes in task settings have led to the more pronounced dominance of the model-based learning system, as compared to other studies in the field. In addition, the task and its parameters do not allow to distinguish between the ability and the willingness/motivation to exert cognitive control: While we found no decline in the application of effortful model-based strategies, our data does not allow to exclude the theoretical possibility the participants’ awareness of their hangover symptoms and/or associated expectations of decreased performance motivated them to exert more effort than during the sober session, thus masking small to medium detrimental effects of alcohol hangover on effortful model-based strategies. 

To the best of our knowledge, this is the first publication that explicitly investigates the arbitration between effortful model-based and computationally less demanding model-free learning in alcohol hangover. Yet still, the finding that hangover does not reduce the ability and/or willingness to invest cognitive effort (despite the experimentally applied sleep restriction and the fact that binge drinking is known to decrease sleep quality [42,44,47,48,49]) adds to the general literature on cognitive hangover effects, where cognitive effects that can be reliably observed during alcohol intoxication or AUD cannot always be reproduced during alcohol hangover [6,10,11,12,17], and are not necessarily modulated or worsened by light hangover-associated sleep impediments [40,44]. Given that there is an ongoing debate on whether or not habitual binge drinking impairs cognitive control functions that require high levels of effort [22,66] and all of our participants had been recruited to engage in binge drinking at least one a month (in order to minimize the risk of severe adverse side effects during experimental intoxication), we ran add-on analyses to investigate the potential effects of alcohol use severity on the investigated measures at both sessions. Of note, none of these analyses provided evidence for an association between alcohol use severity (indicated by AUDIT scores) and changes in any of the measures relevant to the arbitration between model-based and model-free behavior. Of note, this finding is in line with a study by Doñamayor et al. [21], who compared both female and male binge drinkers (mean AUDIT score of 16) to healthy controls (mean AUDIT score of 5) of similar age as our sample. While they reported a shift from goal-directed behavior to habitual behavior in binge drinkers, they found no statistical relationship between AUDIT scores, the weighting parameter, model-free scores, or model-based scores across the entire sample, even though they had a similar overall mean and greater variance in AUDIT scores across the entire sample [21]. It also matches reports by Patzelt et al. [67], who found no correlation between alcohol use (as assessed with the AUDIT) and model-based scores in over 900 adult Amazon Mechanical Turk participants.

Given that we only investigated young healthy males, it should be critically discussed whether the null finding reported in this study would also have been found females, or in other age groups. Females tend to metabolize alcohol more slowly than males [68] and have been suggested to report greater subjective hangover symptoms than males [69,70,71]. Lastly, women have been suggested to show greater cognitive impairments than males in case of regular binge drinking [22] and alcohol abuse [72]. Given that women might hence be more vulnerable to the negative cognitive effects of alcohol, our results might unfortunately not be readily generalizable to female populations, thus necessitating further studies. Furthermore, it has been shown that general cognitive and executive functions (e.g., processing speed or working memory) interact with model-based learning [62,73]. It could hence be possible that the typically high functioning levels found in young healthy samples protected our participants from detrimental effects of intoxication. Given that old age has repeatedly been associated with decreased cognitive functions in various domains, including model-based decision making [74,75], and further given that hangover severity might also differ with age [76,77], it could be conceivable that the combination of reduced cognitive resources and altered alcohol hangover might render elderly individuals more vulnerable towards the potential detrimental effects of alcohol on model-based processing. It should therefore be investigated whether our null finding can be reproduced in older samples as well. Lastly, we did not control for factors such as reward sensitivity or the subjective cost of control, which may depend on both internal and external factors. Correcting for the expected value of control as suggested by Shenhav et al. [78] (e.g., controlling for aspects like reward sensitivity, task difficulty, or anterior cingulate cortex activation) might potentially provide new insights and/or help identify functional subgroups. In line with this, it would also have been interesting to assess whether subjective ratings of motivation and invested effort differed between the sober and hungover appointment and/or whether they correlated with any of the assessed parameters.

## 5. Conclusions

In summary, we investigated whether alcohol hangover shifts decision making strategies from a more model-based to a more model-free approach. We asked *n* = 25 young healthy male social drinkers to perform a two-step decision-making task once while sober and once while hungover. Behavioral and modeling parameters were compared across appointments. The lack of significant hangover effects and the positive Bayesian evidence for the null hypothesis in all but one investigated parameters suggest that alcohol hangover, which results from a single binge drinking episode, does not impair the application of effortful and computationally costly model-based learning strategies and/or increase model-free learning strategies. While this finding still awaits confirmation in females and other age groups, it adds to a growing body of literature suggesting that behavioral deficits observed in at-risk drinkers [20,21,25] might not be a mere consequence of alcohol consumption alone [20,23,24,30,55,67]. When applying this finding to a clinical context, it suggests that the behavioral and psychological changes that have been associated with problematic drinking patterns like binge drinking [21,22] and shown to drive and maintain alcohol use disorders (AUD) [16,23,24,25], are not likely to arise as a consequence of hangover (alone).

## Figures and Tables

**Table 1:** Symptom severity ratings on both appointments.

Item	Sober	Hungover	*p*
Overall hangover severity	0.167 ± 0.637	3.640 ± 2.119	<0.001
Regret	0.000 ± 0.000	0.440 ± 1.261	0.039
Headache	0.240 ± 0.831	2.600 ± 2.769	0.001
Sensitivity to light	0.040 ± 0.200	1.680 ± 2.076	0.001
Concentration problems	0.440 ± 0.961	3.640 ± 2.464	<0.001
Clumsy	0.080 ± 0.400	2.120 ± 1.716	<0.001
Confusion	0.000 ± 0.000	1.120 ± 1.166	0.001
Dizziness	0.040 ± 0.200	2.400 ± 2.380	<0.001
Anxiety	0.080 ± 0.277	0.560 ± 0.961	0.020
Depression	0.000 ± 0.000	0.640 ± 1.497	0.008
Apathy	0.120 ± 0.440	1.400 ± 1.780	0.004
Stomach pain	0.120 ± 0.440	0.480 ± 1.229	0.129
Nausea	0.160 ± 0.800	1.520 ± 1.828	0.001
Vomiting	0.040 ± 0.200	0.800 ± 1.384	0.011
Reduced appetite	0.240 ± 1012	1.440 ± 2.022	0.021
Thirst	0.440 ± 1083	3.840 ± 2.267	<0.001
Heart pounding	0.160 ± 0.554	1.280 ± 1.768	0.003
Heart racing	0.000 ± 0.000	0.400 ± 0.707	0.015
Shivering	0.080 ± 0.400	1.083 ± 1.176	0.001
Weakness	0.040 ± 0.200	2.480 ± 2.084	<0.001
Sweating	0.080 ± 0.277	0.920 ± 1.552	0.004
Tired	0.560 ± 1083	4.080 ± 2.448	<0.001
Sleepiness	0.440 ± 0.961	3.680 ± 2.410	<0.001
Sleeping problems	0.120 ± 0.332	0.720 ± 1.242	0.036

Average ± SD rating of each symptom on a Likert-scale ranging from 0 (no symptoms) to 10 (extreme symptoms), as suggested by van Schrojenstein Lantman et al. [4,50]. Participants had been asked to rate each item on both appointments, irrespective of whether or not they had consumed alcohol the night before the sober appointment and also irrespective of whether they attributed a given complaint to alcohol hangover. Whenever the average rating was greater than zero on both appointments, the appointments were compared using paired Wilcoxon signed-rank tests. Whenever all of the ratings in the sober session were zero, the hungover appointment was compared to zero using one sample Wilcoxon signed-rank tests. Uncorrected *p*-values of the conducted tests are given in the right column.

**Table 2:** Descriptive task statistics for the two-step decision-making task for the sober and hangover session.

	Mean	SEM	SD	Min	Max
*sober*					
MF-score	0.08	0.04	0.18	−0.25	0.39
MB-score	0.50	0.07	0.33	−0.10	1.10
Final score	357.12	24.90	124.50	66	564
First stage RT	491	28	141	136	728
Second stage RT	585	27	136	198	886
*hangover*					
MF-score	0.06	0.04	0.18	−0.28	0.35
MB-score	0.49	0.05	0.25	<−0.01 *	0.98
Final score	361.48	19.34	96.71	180	521
First stage RT	489	24	121	204	617
Second stage RT	597	12	62	505	762

MF-score: model-free score; MB-score: model-based score; final score: accumulated outcomes at the end of the task (in points); RT: reaction time in msec. * The true value lies between −0.01 and 0.00.

**Table 3:** Distribution of estimated parameters based on the hybrid dual-system reinforcement-learning model for the sober and hangover session.

Percentile	ω	α	β	λ	π
*sober*					
25	0.70	0.81	3.39	0.00	0.11
50	0.83	0.89	4.70	0.48	0.16
75	0.90	1.00	5.48	0.84	0.19
*hangover*					
25	0.68	0.75	3.16	0.28	0.09
50	0.88	0.86	4.04	0.51	0.20
75	0.95	0.98	5.52	0.79	0.23

The weighting parameter ω represents the balance between model-based (ω > 0.5) and model-free learning (ω < 0.5). The learning rate α indicates to what extent new information is incorporated in the Q-value update. The inverse temperature β determines the randomness of decision-making. The decay-rate parameter λ represents the degree to which experience in later stages influences first stage Q-value update. The choice stickiness parameter π indicates perseveration tendencies (π > 0).

**Table 4:** Correlation between AUDIT and two-step task performance in the sober and hangover session.

	*r* (*p*)	BF	τ (*p*)
*sober*			
MF-score	0.24 (0.24)	3.26	0.15 (0.32)
MB-score	0.21 (0.32)	3.94	0.16 (0.28)
Final score	0.30 (0.15)	2.35	0.21 (0.17)
ω	0.21 (0.31)	3.88	0.16 (0.28)
π	0.06 (0.76)	6.21	−0.05 (0.74)
*hangover*			
MF-score	<−0.01 * (0.98)	6.50	0.03 (0.85)
MB-score	0.16 (0.45)	4.90	0.13 (0.37)
Final score	−0.11 (0.60)	5.67	−0.14 (0.34)
ω	0.08 (0.72)	6.10	0.11 (0.45)
π	−0.01 (0.95)	6.49	0.03 (0.85)

BF: Bayes Factor; MF-score: model-free score; MB-score: model-based score; final score: accumulated outcomes at the end of the task (in points); weighting parameter ω: balance between model-based (ω > 0.5) and model-free learning (ω < 0.5); Choice stickiness parameter π: indicates perseveration tendencies (π > 0). * The true value lies between −0.01 and 0.00.

## Supplementary Materials

The following are available online at https://www.mdpi.com/2077-0383/9/5/1453/s1: Simulation of Transition and Reward Distribution (including Figures S1 and S2, which display reward distributions), Investigation of Hypothetical Task Order Effects (including Tables S1–S4, which provide descriptive statistics and estimated parameters with respect to hypothetical appointment order effects), a data sheet containing the equation used to determine individually served alcohol amounts and document drinking during experimental intoxication.
