# Supplementary material for: Alcohol Hangover Does Not Alter the Application of Model-Based and Model-Free Learning Strategies
Source: J Clin Med. 2020 May 13;9(5):1453. doi: 10.3390/jcm9051453 (PMC7290484; doi:10.3390/jcm9051453)
Supplement: Supplementary file 1 [file jcm-09-01453-s001.zip › Supplements/SupplementaryInformation_FINAL.pdf]

Supplementary Information for

Alcohol hangover does not alter the application of model-based and model-free learning strategies

Julia Berghäuser, Wiebke Bensmann, Nicolas Zink, Tanja Endrass, Christian Beste and Ann-Kathrin Stock (correspondence: Ann-Kathrin.Stock@ukdd.de)

Simulation of Transition and Reward Distribution

The simulation was performed with scripts provided by Wouter Kool on Github [1] (https://github.com/wkool/tradeoffs/tree/master/simulations) with Matlab 2018b (The MathWorks Inc., Natick, MA, USA).

For each run (100 runs in total), a transition matrix for both first stage stimuli was randomly generated with 80% common and 20% rare transition for 250 trials.
Rewards were independently generated with reflecting boundaries ranging from 0 to 1 and with a Gaussian random walk (M=0, SD=0.2) for the four choice options at the second stage.

Choices were simulated based on the dual-system hybrid learning model for different parameter combinations [1,2]: The weighting parameter omega (range = [0 1], step size = 0.1), the inverse temperature beta (range = [0 10], step size = 1), and learning rate alpha (range = [0 1], step size = 0.1) were simulated, while lambda was fixed to 0.5. Performance was simulated for each parameter combination with 300 iterations. The linear effect between reward rate and omega was estimated and averaged for each parameter combination of alpha and beta. To identify the best suitable reward and transition matrix, we calculated a weighted frequency score of the relationship between reward rate and omega (higher relationships were weighted more strongly than smaller ones). This resulted in one value for each run, which we used to determine the highest relationship between omega and reward rate for the whole parameter space. Figure S1 and Figure S2 show the used reward distributions.

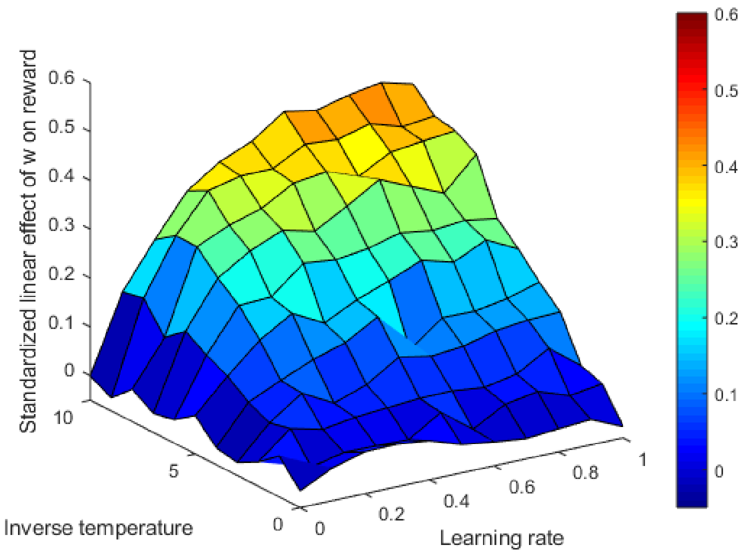

Figure S1. Reward distribution 1: Surface plot of the relationship between weighting parameter ω and the reward rate as a function of inverse temperature β and learning rate α.

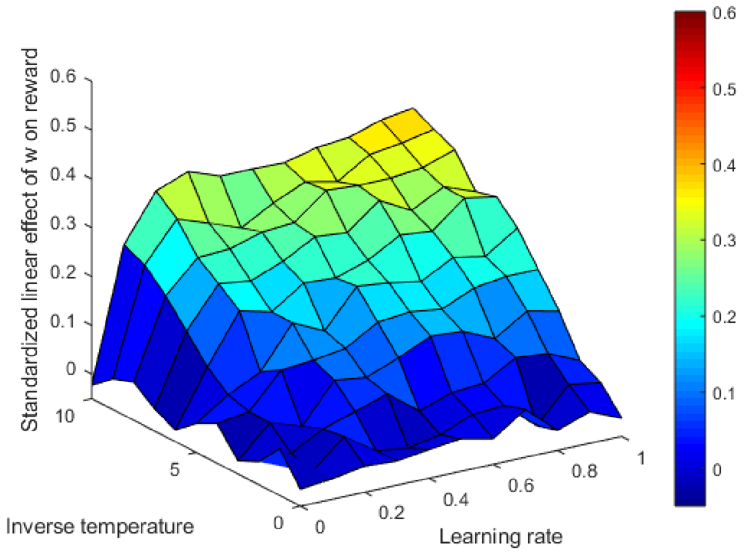

Figure S2. Reward distribution 2: Surface plot of the relationship between weighting parameter ω and the reward rate as a function of inverse temperature β and learning rate α.

Investigation of Hypothetical Task Order Effects

We had decided to control for potential learning/order effects by counterbalancing the order of appointments across the sample so that half of the participants first performed the paradigm sober and then hungover, while the other half first performed the paradigm hungover and then sober. Consequently, potential learning could not have confounded the hangover effect, as such effects would have been averaged out across the two appointment order groups. Furthermore, we randomly varied stimulus positions on the screen for ach trial and employed two different task versions/stimuli on the first (T1) and second appointment (T2) of each participant (i.e., irrespective of whether the first appointment was assessed sober or hungover). While this additional precaution should have made it very unlikely to obtain strong confounding learning effects, we ran additional analyses to check for this. The descriptive data of the behavioral scores are given in Table S1.

Table S1 Descriptive statistics for the two-step decision-making task for both task administrations (irrespective of treatment).

	Mean	SEM	SD	Min	Max
Session T1					
MF-score	0.07	0.03	0.16	–0.28	0.35
MB-score	0.46	0.06	0.30	–0.10	0.98
Final score	271.84	15.64	78.20	66	387
Session T2					
MF-score	0.08	0.04	0.20	–0.28	0.39
MB-score	0.53	0.06	0.28	<0.01	1.10
Final score	446.76	10.61	53.03	355	564

MF-score: model-free score; MB-score: model-based score; final score: accumulated outcomes at the end of the task (in points).

There was no significant difference between T1 and T2 (irrespective of treatment) with respect to the MF-score ($t(24) = –0.26$, $p = 0.80$) and the MB-score ($t(24) = –1.09$, $p = 0.29$). Bayesian analyses indicated positive evidence in favor of the null hypothesis, i.e., the assumption that the MF-score (BF = 6.29) and the MB-Score (BF = 3.70) did not differ between the two task administrations.

Participants earned more in terms of outcome points (final score) at the end of T2 than at the end of T1 ($t(24) = –12.58$, $p < 0.001$). This improvement in overall task performance might however be due

to familiarity with the task settings. Bayesian analysis indicated positive evidence for the alternative hypothesis (BF < 10^{-10}), i.e., differences in the final scores between T1 and T2.

Descriptive data of the estimated parameters are given in Table S2.

Table S2 Distribution of estimated parameters based on the hybrid dual-system reinforcement-learning model for T1 and T1 (irrespective of treatment).

Percentile	ω	π
Session T1		
25	0.63	0.07
50	0.85	0.19
75	0.94	0.24
Session T2		
25	0.77	0.12
50	0.86	0.16
75	0.95	0.20

The weighting parameter ω represents the balance between model-based ($\omega > 0.5$) and model-free learning ($\omega < 0.5$). The choice stickiness parameter π indicates perseveration tendencies ($\pi > 0$).

There was no significant difference between T1 and T2 (irrespective of treatment) with respect to the weighting parameter ω ($t(24) = -0.98$, $p = 0.34$; $Z = -1.39$, $p = 0.17$) and the choice "stickiness" parameter π ($t(24) = -0.54$, $p = 0.60$; $Z = -0.34$, $p = 0.74$). Bayesian analyses for ω (BF = 4.11) and π (BF = 5.65) provided positive evidence for the null hypotheses, i.e., both parameter did not differ between T1 and T2.

Additionally, we calculated Δ scores (T2 minus T1) to characterize potential improvements due to repeated task administration (see Tables S3 and S4). Participants who started with the sober session and those who started with the hangover session were compared with independent sample t-tests and additional Bayesian analyses (see Table S5). Furthermore, we conducted non-parametric analyses (Mann-Whitney-test) due to the small sample size per group.

Table S3 Descriptive statistics for the two-step decision-making task for both appointment order groups (irrespective of treatment).

	Mean	SEM	SD	Min	Max
Sober first group (n = 12)					
ΔMF-score	–0.01	0.08	0.28	–0.38	0.60
ΔMB-score	0.05	0.08	0.28	–0.37	0.53
ΔFinal score	186.75	16.79	58.15	113	323
Hangover first group (n = 13)					
ΔMF-score	0.03	0.07	0.25	–0.45	0.30
ΔMB-score	0.08	0.10	0.34	–0.62	0.57
ΔFinal score	164.00	22.00	79.33	20	294

Δ: difference score between T2 and T1; MF-score: model-free score; MB-score: model-based score; final score: accumulated outcomes at the end of the task (in points).

There was no significant difference between subjects who started with the sober appointment and subjects who started with the hangover appointment for either the ΔMF-score ($t(23) = 0.37$, $p = 0.72$; $Z = -.76$, $p = 0.47$) or the ΔMB-score ($t(23) = 0.21$, $p = 0.83$; $Z = -0.44$, $p = 0.69$). Bayesian analyses for the ΔMF-Score (BF = 3.34) and the ΔMB-score (BF = 3.47) provided positive evidence for the null hypothesis, i.e., that appointment order does not influence performance changes due to task repetition. Furthermore, we found no order group differences in the improvement in cumulative points at the end of the task (Δfinal score) ($t(23) = -0.81$, $p = 0.43$; $Z = -0.60$, $p = 0.57$). Bayesian analysis provided weak evidence for the assumption that improvement in cumulative points did not differ between subjects who started with the sober appointment and subjects who started with the hangover appointment (BF = 2.69).

Table S4 Distribution of estimated parameters based on the hybrid dual-system reinforcement-learning model for both appointment order groups (irrespective of treatment).

**Percentile**	**Δω**	**Δπ**
Sober first group (n = 12)		
25	0.03	–0.06
50	0.10	0.03
75	0.27	0.14
Hangover first group (n = 13)		
25	–0.10	–0.08
50	–0.04	<–0.01*
75	0.25	0.04

Δ: difference score between T2 and T1. The weighting parameter ω represents the balance between model-based (ω > 0.5) and model-free learning (ω < 0.5). The choice stickiness parameter π indicates perseveration tendencies (π > 0). *The true value lies between –0.01 and 0.00.

There was also no significant difference between appointment groups for the weighting parameter Δω ($t(23) = –0.52$, $p = 0.61$; $Z = –1.47$, $p = 0.15$) and the choice "stickiness" parameter Δπ ($t(23) = –1.43$, $p = 0.17$; $Z = –1.41$, $p = 0.17$). Bayesian analyses provided positive evidence for the null hypothesis for Δω (BF = 3.16) and weak evidence for Δπ (BF =1.54), that is, appointment order does not modulate performance change due to task repetition.

References

1. Kool, W.; Cushman, F.A.; Gershman, S.J. When Does Model-Based Control Pay Off? *PLoS Comput. Biol.* **2016**, *12*, e1005090, doi:10.1371/journal.pcbi.1005090.
2. Daw, N.D.; Gershman, S.J.; Seymour, B.; Dayan, P.; Dolan, R.J. Model-based influences on humans' choices and striatal prediction errors. *Neuron* **2011**, *69*, 1204–1215, doi:10.1016/j.neuron.2011.02.027.
